# Incidental dose distribution to locoregional lymph nodes of breast cancer patients undergoing adjuvant radiotherapy with tomotherapy - is it time to adjust current contouring guidelines to the radiation technique?

**DOI:** 10.1186/s13014-019-1328-7

**Published:** 2019-08-01

**Authors:** Michael Mayinger, Kai Joachim Borm, Constantin Dreher, Hendrik Dapper, Marciana-Nona Duma, Markus Oechsner, Severin Kampfer, Stephanie Elisabeth Combs, Daniel Habermehl

**Affiliations:** 1 Department of Radiation Oncology, Klinikum rechts der Isar, Technical University Munich, Ismaninger Str. 22, D-81675 Munich, Germany; 2 Department of Radiation Oncology, University Hospital Zurich, University of Zurich, Rämistrasse 100, CH-8091 Zurich, Switzerland; 3 Institute of Innovative Radiotherapy (iRT), Helmholtz Zentrum München, Ingolstädter Landstraße 1, D-85764 Oberschleißheim, Germany; 4 Deutsches Konsortium für Translationale Krebsforschung (DKTK), Partner Site Munich, Munich, Germany

**Keywords:** Tomotherapy, Breast cancer, Contouring guidelines, Incidental dose, Locoregional lymph nodes, Adjuvant radiotherapy

## Abstract

**Purpose/objective(s):**

Along with breast-conserving surgery (BCS), adjuvant radiotherapy (RT) of patients with early breast cancer plays a crucial role in the oncologic treatment concept. Conventionally, irradiation is carried out with the aid of tangentially arranged fields. However, more modern and more complex radiation techniques such as IMRT (intensity-modulated radio therapy) are used more frequently, as they improve dose conformity and homogeneity and, in some cases, achieve better protection of adjacent risk factors. The use of this technique has implications for the incidental- and thus unintended- irradiation of adjacent loco regional lymph drainage in axillary lymph node levels I-III and internal mammary lymph nodes (IMLNs). A comparison of a homogeneous “real-life” patient collective, treated with helical tomotherapy (TT), patients treated with 3D conformal RT conventional tangentially arranged fields (3DCRT) and deep inspiration breath hold (3DCRT-DIBH), was conducted.

**Materials/methods:**

This study included 90 treatment plans after BCS, irradiated in our clinic from January 2012 to August 2016 with TT (*n* = 30) and 3D-CRT (*n* = 30), 3DCRT DIBH (*n* = 30). PTVs were contoured at different time points by different radiation oncologists (> 7). TT was performed with a total dose of 50.4 Gy and a single dose of 1.8 Gy with a simultaneous integrated boost (SIB) to the tumor cavity (TT group). Patients irradiated with 3DCRT/3DCRT DIBH received 50 Gy à 2 Gy and a sequential boost. Contouring of lymph drainage routes was performed retrospectively according to RTOG guidelines.

**Results:**

Average doses (DMean) in axillary lymph node Level I/Level II/Level III were 31.6 Gy/8.43 Gy/2.38 Gy for TT, 24.0 Gy/11.2 Gy/3.97 Gy for 3DCRT and 24.7 Gy/13.3 Gy/5.59 Gy for 3DCRT-DIBH patients. Internal mammary lymph nodes (IMLNs) Dmean were 27.8 Gy (TT), 13.5 Gy (3DCRT), and 18.7 Gy (3DCRT-DIBH). Comparing TT to 3DCRT-DIBH dose varied significantly in all axillary lymph node levels and the IMLNs. Comparing TT to 3DCRT significant dose difference in Level I and IMLNs was observed.

**Conclusion:**

Dose applied to locoregional lymph drainage pathways varies comparing tomotherapy plans to conventional tangentially arranged fields. Studies are warranted whether dose variations influence loco-regional spread and must have implications for target volume definition guidelines.

## Introduction

Radiation therapy (RT), with or without a boost to the surgical bed, has a crucial role in the adjuvant treatment of early breast cancer by improving local control and is advocated in national and international treatment recommendations [1, 2]. Overall survival (OS) benefit of adjuvant RT for breast cancer patients is well established [3–5]. However, radiation therapy in these studies was delivered to the breast and to all corresponding lymphatic drainage regions, including the axilla, supraclavicular fossa, and internal mammary lymph nodes (IMLNs).

Today in patients with early stage tumors, no further treatment of the axilla is indicated after sentinel node biopsy [6].

Nonetheless, recently, results of two randomized trials have shown reduced rate of breast-cancer recurrence, improved disease-free survival and distant disease-free survival after irradiation of the locoregional nodal drainage system in lymph-node positive patients and node-negative patients with risk factors [7, 8]. Poortmans also reported a small benefit on overall survival [8].

A study by Thorsen et al. reported a 3.7% 8-year OS benefit when treating the IMLNs of all patients with right-sided disease compared to left-sided disease where IMLNs were not treated [9].

However, higher pulmonary toxicity, risk of frozen shoulder syndrome is expected when the axillary lymph nodes and the IMLNs are included in the RT target volume. Therefore, loco regional lymph drainage routes irradiation is still controversial.

The RT target volumes are well defined for conventional conformal three-dimensional (3D) RT techniques [10]. When the 3D tangential field (TF) technique is used, incidental irradiation of loco regional lymph drainage routes –especially the axillary level I and II- is usually accepted although they are not included in the target volume. Published analyses of adequate dose coverage of the axillary level I and II after TF-RT are heterogenous and partially contradictory [11–15].

Also, deep inspiration breath hold (DIBH) radiation therapy gains more popularity for breast cancer therapy, as it results in lower cardiac doses [16]. Due to the deep inspiration breath hold, several anatomical changes occur. Tomotherapy Hi-Art II system (Accuray Inc., Sunnyvale, CA) is a RT platform able to deliver highly conformal intensity modulated radiotherapy (IMRT) plans within a helical geometry under image guidance (IGRT). It combines continuous rotation of the beam delivery gantry, concomitant couch translation (along the craniocaudal direction) and MLC modulation. Tomotherapy therefore is particularly suitable for breast irradiation [17, 18]. With even more conformal tomotherapy irradiation the incidental dose to the loco regional lymph drainage routes might be different.

Therefore, the purpose of this study was to evaluate the incidental irradiation of adjoining loco-regional lymph drainage routes (axillary lymph node levels I-III and IMLN) with no formal indication for irradiation of the regional lymph drainage routes in a real-life cohort. Patients were treated with: helical IMRT on a tomotherapy (TT) accelerator, 3D conventional tangentially arranged fields (3D) or with a gating technique using conventional tangentially arranged fields (3DCRT-DIBH) with Deep-Inspiration Breathhold (DIBH).

## Methods

### Participants

This retrospective study included 60 female patients treated with either tangential field (TF) 3D conformal radiotherapy (3DCRT), DIBH or helical tomotherapy. All patients underwent surgery and treatment between January 2012 and August 2016 and were candidates to postoperative irradiation of breast and without regional node irradiation. Patients’ characteristics were evaluated including the site of treatment, tumor stage, and type of surgery (breast-conserving surgery, or mastectomy with immediate reconstruction) from the institutional database (MiRO-Database) and are shown in Table 1. The study was approved by the Local Ethics Commission of the Medical Faculty of the Technical University of Munich (TUM), Klinikum rechts der Isar.

**Table 1:** Dose constraints employed during the planning process

	TT	3DCRT /3DCRT-DIBH
Mean Age Tumor stage	55.5 (± 5.8)	61.6 (± 9.4)
pTis	0	4
pT1a	1	4
pT1b	9	6
pT1c	15	9
pT2	5	1
PT3	0	1
ypT0	0	5
Tumor quadrant		
Upper outer	16	14
Upper inner	4	5
Retro/perimammillary	6	5
Lower outer	3	4
Lower inner	1	2
Type of surgery		
BCS	30	29
mastectomy (immediate reconstruction)	0	1
Boost	simultaneous	Sequential
Boost dose	28 × 2.25 Gy	5 × 2 Gy (*n* = 4) 8 × 2 Gy (*n* = 26)
Site of treatment		
left	15 (50%)	30 (100%)
right	15 (50%)	0 (0%)

### Treatment and volume delineation

Patients were immobilized in supine position with a wing board. Thirty patients were irradiated with the tomotherapy system (Accuray, Sunnyvale, US). The other 30 patients underwent DIBH as well as FB (free breathing) treatment planning from 2012 to 2014. The patients were treated with DIBH if the heart Dmean dose was higher than 3Gy in the FB radiotherapy treatment plan - according to institutional guidelines [19]. Total prescribed dose was 50.0 Gy or 50.4 Gy, in conventional fractions of 2.0 or 1.8 Gy/day, for all the FB and FB treatment plans. Patients treated with helical tomotherapy received a simultaneous irradiation boost to the surgical bed with single doses of 2.25 up to 63 Gy. The 3DCRTand 3DCRT-DIBH patient all received sequential boost radiotherapy which was not considered in this study. The PTVs (planning target volumes) were defined according to the available evidence at a given time by several differnet radiation oncologists (> 7). All treatment plans were optimized in order to achieve the best possible dose distribution. In both techniques, the goal was improved dose homogeneity in the target volume ranging from − 5 to + 7%, in accordance with the ICRU 50 recommendations [20]. Wedges and field-in-field strategies were used. Dose constraints employed during the planning process are depicted in Table 2.

**Table 2:** Dose constraints employed during the planning process

Organ at risk	Dose constraint
Ipsilateral lung	V20 < 20%
Contralateral lung	mean < 5 Gy
Heart	V30 < 10%, mean < 3 Gy
Contralateral Breast	mean < 5 Gy
Myelon	< 45 Gy
Ipsilateral Humerus	Dmax ≤100% of prescribed dose

**Fig. 1:**
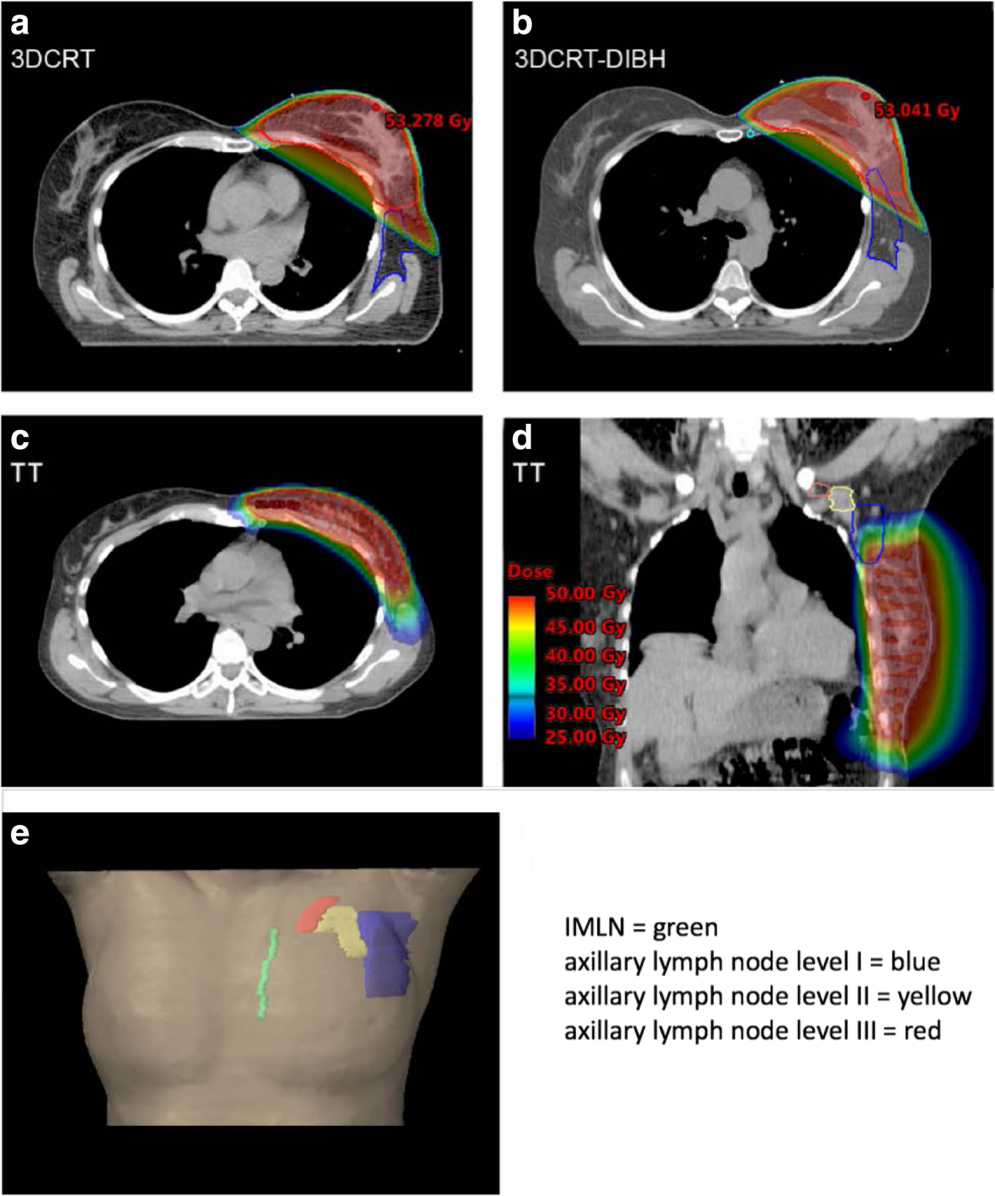
shows dose distributions from 25 Gy to 50 Gy for: 3DCRT (**a**), 3DCRT-DIBH (**b**) and tomotherapy (**c**, **d**) and anatomic locations of axillary lymph node levels and the IMLN (**e**)

To compare incidental doses to non-target tissues we contoured the axillary lymph node levels and the internal mammary lymph nodes (IMLNs) following the Radiation Therapy Oncology Group [21] recommendations (Fig. 1): https://www.rtog.org/LinkClick.aspx?fileticket=vzJFhPaBipE=&tabid=236).

### Statistical analysis of radiation dose volume relations

DVHs (dose volume histograms) were exported in text format using Eclipse Treatment Planning System (Version 13.0, VARIAN, Palo Alto, US). They were then imported into R (Version 3.3.2., R Foundation for Statistical Computing, Vienna, Austria) and processed using the DVHmetrics library to generate dose volumes and dose volume histograms. DVH metrics were then further analyzed using two-sided, non-paired t tests in GraphPad Prism (GraphPad Prism version 6.00, GraphPad Software, San Diego, California). A *p* < 0.05 was considered statistically significant.

## Results

Ninety treatment plans were generated of 60 patients who underwent breast surgery and 3D RT between January 2012 and March 2016 using the lymph node volumes according to the RTOG guidelines. For all 30 patients treated with 3DCRT, further 30 3DCRT-DIBH plans were generated to evaluate possible heart dose reduction of 3DCRT-DIBH resulting in 60 treatment plans. For all TT patients only one IMRT plan was generated. The mean dose (Dmean) in the axillary lymph node level I was 31.6 Gy (±13.7 Gy), 24.0 Gy (±10.1 Gy) and 24.7 Gy (±10.5Gy) for TT, 3DCRTand 3DCRT-DIBH patients, respectively. For Level II doses were 8.43 Gy (±7.34 Gy), 11.2 Gy (±9.65 Gy) and 13.3 Gy (±5.59 Gy) in TT, 3DCRT and 3DCRT-DIBH patients, respectively. For Level III mean doses were 2.38 Gy (±2.58 Gy), 3.97 Gy (±6.00 Gy) and 5.59 Gy (±6.9 Gy) for patients in the TT-, 3DCRTand 3DCRT-DIBH group, respectively (Table 3). In summary, the TT technique leads to clearly increased doses to level I (32 and 28% higher than in 3DCRT and 3DCRT-DIBH patients, respectively. Nevertheless, for the axillary levels II and III 3DCRT-DIBH patients experienced the highest dose compared to TT- (56 and 235% increased dose for level II and III, respectively) and 3D-patients (119 and 141% increased dose for level II and III, respectively). Comparing TT to 3DCRT patients, a significant difference in axillary lymph node level I (*p* ≤ 0.01) was observed. Nonetheless, 16 of the TT patients had a tumor in the outer upper quadrant and were treated with a SIB. There was a significant difference comparing TT to 3DCRT-DIBH group in all axillary lymph node levels (Level I: *p* ≤ 0.03; Level II: *p* ≤ 0.03; Level III: *p* ≤ 0.02). There was no significant difference comparing 3DCRTpatients to 3DCRT-DIBH in the axillary lymph node. No significant dose differences were observed comparing left and right sided axillary lymph nodal levels within the tomotherapy group (Table 4).

**Table 3:** Dose constraints employed during the planning process

	TT (mean dose in Gy, ±SD)	3DCRT (mean dose in Gy, ±SD)	3DCRT-DIBH (mean dose in Gy, ±SD)
Level I			
Dmean	31.6 (± 13.5)	24.0 (± 10.1)	24.6 (± 10.5)
Dmedian	33.3 (± 15.4)	21.5 (± 15.7)	23.8 (± 15.6)
V45	30.0 (± 20.8)	36.8 (± 16.3)	35.4 (± 23.2)
V40	43.2 (± 25.7)	44.5 (± 19.0)	43.4 (± 25.6)
V25	68.8 (± 32.7)	54.3 (± 21.5)	52.0 (± 27.2)
Level II			
Dmean	8.4 (±7.3)	11.2 (± 9.7)	13.3 (± 5.6)
Dmedian	7.4 (±7.6)	7.3 (± 12.3)	10.5 (± 12.0)
V45	0 (± 0)	8.7 (± 11.3)	14.8 (± 25.7)
V40	0.4 (± 1.5)	16.7 (± 16.1)	24.0 (± 28.5)
V25	8.7 (± 15.4)	25.6 (± 22.7)	32.6 (± 31.7)
Level III			
Dmean	2.4 (± 2.6)	4.0 (± 6.1)	5.6 (± 6.9)
Dmedian	1.6 (± 1.5)	2.7 (± 5.6)	3.6 (± 6.7)
V45	0 (± 0)	1.5 (± 4.2)	9.2 (± 24.7)
V40	0.0 (± 0.1)	3.5 (±7.6)	12.6 (± 25.8)
V25	1.0 (± 3.2)	7.2 (± 13.3)	16.7 (± 28.4)
ILMLN			
Dmean	27.9 (± 8.0)	13.5 (± 10.8)	18.7 (± 8.0)
Dmedian	29.6 (± 9.1)	19.0 (± 16.1)	12.4 (± 14.2)
V45	9.3 (± 15.2)	7.4 (± 13.4)	12.0 (± 16.8)
V40	19.8 (±22.7)	13.2 (±20.4)	21.1 (± 24.8)
V25	59.1 (± 28.7)	22.7 (± 26.2)	33.6 (± 30.6)
Heart left sided RT			
Dmean	3.8 (± 1.2)	2.8 (± 1.7)	1.1 (± 0.4)
V30	0.1 (± 0.2)	2.9 (± 2.7)	0.2 (± 0.4)
V20	0.4 (±0.7)	3.9 (± 3.8)	0.3 (± 0.6)
Lung (ipsilateral)			
Dmean	9.1 (± 1.5)	7.7 (± 1.8)	6.8 (± 1.6)
V30	6.9 (± 2.4)	11.9 (± 3.3)	10.0 (± 2.9)
lV20	12.6 (± 3.7)	14.3 (± 4.0)	12.0 (± 3.3)

*TT* Tomotherapy, *3DCRT* conventional tangentially arranged fields, *3DCRT-DIBH* breathing gated conventional tangentially arranged fields

**Table 4:** Dose constraints employed during the planning process

Nodal level	Mean dose right side TT	Mean dose left side TT	*p* value
Nodal level 1	26.34 Gy	36.77 Gy	0.06
Nodal level 2	6.37 Gy	10.49 Gy	0.08
Nodal level 3	1.57 Gy	3.19 Gy	0.11

For the IMLNs Dmean was 27.8 Gy (± 8.0Gy), 13.5 Gy (± 10.8 Gy) and 18.7 Gy (± 11.7 Gy) for TT, 3DCRTand 3DCRT-DIBH patients. Therefore, irradiation using the tomotherapy technique lead to an increase of the IMN dose of 206 and 149% compared to the 3DCRT- or the 3DCRT-DIBH-technique, respectively. Differences between the TT- and the 3DCRT- as well as the 3DCRT-DIBH-technique were statistically significant (*p* ≤ 0.001). Additionally, there were no statistically significant differences between the two conventional technique groups.

Heart Dmean for left sided treatment plans were 3.8 Gy (± 1.2 Gy), 2.8 Gy (± 1.7 Gy) and 1.1 Gy (± 0.4 Gy) in the TT-, 3DCRTand 3DCRT-DIBH group, respectively. Only left sided treatment plans were analyzed as heart doses strongly vary comparing right and left treatment plans. Ipsilateral lung Dmean in the TT-, 3DCRT and 3DCRT-DIBH group 9.1 Gy (± 1.5 Gy), 7.7 Gy (± 1.8 Gy) and 6.8 Gy (± 1.6 Gy).

## Discussion

Our analysis shows that the dose applied to the locoregional lymph drainage pathways varies significantly between tomotherapy and conventional 3D-RT-techniques. The study shows that a lower dose is delivered to level II and level III when using the tomotherapy technique. However, doses applied to the IMLNs and axillary level I lymph node chain were significantly higher using tomotherapy.

Through IMRT inverse planning, tomotherapy actively relocates isodoses from organ-at-risk areas (e.g. contralateral breast, lungs, heart) towards areas such as the ILMLN and axillary level I region where usually are no restrictions or constraints for the treatment plan optimization. Tomotherapy delivered a higher dose to level I (Fig. 1). Further, 16 of the 30 patients had an upper outer quadrant tumor in the TT group that was treated with SIB. This definitely plays a role in the dose in Level I and might be one of the limitations of this study as it was compared to 50 Gy whole breast 3D-CRT. Further comparison of simultaneous and sequential boost may not be fully accurate.

This dose modelling, instead of the more rigid geometry observed using tangential irradiation, most likely causes the observed differences in dose deposition to the ILMLNs.

Published literature to date reveals disparity in axillary fields (especially cranial border) and axillary doses; only few studies revealed an adequate coverage with 3DCRT and high tangents [11–15]. Krasin et al. reconstructed 2D plans for 25 patients on 3D planning system and analyzed dose-volume data. Mean axillary doses were 32 Gy, 26 Gy and 18 Gy for levels I, II and III, respectively [22]. Alco et al. evaluated dose coverage of axillary volumes with high tangents. Reported doses were 39.4 Gy in level I and 26.6 Gy in level II. Using high tangents modified with multi-leaf collimators (MLC) the surrounding isodose was increased to 49.8 Gy and 47 Gy, respectively [13].

The Skagen Trial 1 reported a D95% of 85% in nodal levels, and D95% of 49% in the ILML [23].

Aristei et al. examined dose distributions of tangential RT plans and observed median doses of 38.6 Gy in level I and 20.6 Gy in level II [24]. UK IMPORT LOW showed that partial breast irradiation with 40,05 Gy and thus delivering only 36 Gy to the total breast is equally effective as total breast irradiation [10]. Borm et al. observed a significant dose reduction in level I of 3DCRT-DIBH plans compared to 3DCRT plans [25]. In this previous study, the authors standardized the PTVs to rule out the impact of interobserver variability. In the current study, since different patient collectives (TOMO vs. Tangential field irradiation), treated over a period of 4 years (2012 to 2016) were analyzed, standardization of PTVs was waived. We decided to focused on the actual treatment plans used in clinical practice over a long period of time. This accounts for the differences observed between the studies regarding the effect of DIBH on tangential field irradiation and is a limitation of our study.

Incidental axillary doses achieved by IMRT have not been thoroughly investigated in literature, despite the widespread use of this technique for breast irradiation [26–29]. Doses delivered to axilla in the present study match those in studies using high tangents. Our results confirm findings of previous studies that axilla may not be adequately covered in treatment plans designed to treat breast but 3DCRT is coincidentally treating a significant portion of axilla [30].

Whether the incidental axillary dose is adequate as a prophylactic therapy for microscopically positive axilla and whether the raise of the cranial border of the field needs to be performed in cases with sentinel lymph node biopsy (SLNB) alone to intentionally enable higher axillary coverage, are interesting but to date remain unanswered. It is plausible that a dose less than 95% of the prescription dose is adequate to treat microscopic axillary disease. Wither et al. suggested a shallow dose response curve for microscopic disease. Although doses of 45–50 Gy are more relevant for sterilization of subclinical disease, it is possible that a dose in the range of 30 Gy may be capable of some regional control [31]. Low doses (10–30 Gy) have been reported to sterilize microscopic tumor in ovarian, bladder and breast carcinomas and should not be neglected [32]. Withers et al. suggest that that noteworthy benefit is achieved by doses as low as 14–21 Gy, if delivered close to the treatment of primary [33]. Especially in adjuvant setting lower doses possibly still have strong therapeutic effects.

In low burden axilla, post-SLNB, complete axillary lymph node dissection and adjuvant radiation to breast alone studies found good control rates [6, 34, 35]. Majority of those studies have used 3D-therapy, not IMRT or tomotherapy. During clinical decision making as well as when defining novel prospective trials, one should keep in mind that different radiotherapy techniques can have a significant impact on dose distributions outside the target volumes. For breast radiotherapy, this is especially important for the lymphatic regions, where the present study clearly shows that modern IMRT with tomotherapy delivers lower incidental radiation to axillary levels II and III as compared to classical 3D-radiotherapy. Dose delivered to the axilla during tomotherapy treatment for early breast cancer has not been considered, compared to the cases of advanced disease where the axilla was included in the target volume. However, this carries the potential risk of missing opportunity for regional control of occult metastasis of the axilla, especially for patients with limited positive sentinel lymph nodes.

Relevant clinical trials, which established adjuvant RT regularly used 3DCRT and 3DCRT-DIBH. Using breathing adapted radiotherapy, 3DCRT-DIBH allows for temporary, reproducible immobilization of internal thoracic structures [36]. Breathing adapted radiotherapy monitors the patient’s breathing cycle and implements a breath hold at a predefined lung volume level. This maximizes the distance between chest wall and heart and results in a reduction of irradiated cardiac volume and dose, for some patients [36]. Nowadays, new techniques such as IMRT / tomotherapy are also frequently being used. Advantages of this are higher conformity and partial better protection of OARs (organs at risk) especially in patients with special anatomy (funnel breast, etc.). Disadvantage is that exact consequences for treatment response remain unknown, as there are no relevant studies comparing treatment success rates. Higher dose on the ILMLNs and increased dose on the axilla level I may be beneficial [37]. Besides, theoretical side effects must be considered, as well as the fact that comparing conventional fractionation to hypofractionated schedule in terms of dose contribution to the nodes is not completely accurate. Therefore, the question how constraints should be set remains: Are increased isodoses in the axilla beneficial? Are dose constraints for the loco regional lymph nodes necessary? Should tomotherapy inverse planning consider incidental dose distribution? Contouring atlases are valuable, but tangential irradiation is not conformal and places a very high amount of dose (> 90% isodose) outside the PTV. This is accepted, but only because better loco regional control and dose distribution is expected. Tailored RT for individual patients might be needed. Refinement/size adjustment of the PTV may be required for IMRT to obtain similar dosimetry as in 3DCRT.

Thus, future studies are warranted investigating a potential influence of the dose deviations overserved on loco-regional spread. And – based on the present data – radiation oncologists as well as other disciplines must be aware that radiation therapy remains a highly individualized treatment that not only includes total dose and fractionation, but impact of treatment technique, anatomical variations as well as a series of other patient-related factors. Therefore, every treatment plan is more than a single and quick decision in interdisciplinary conferences, and requires extensive and intricate knowledge and diligence.

## Conclusion

The dose applied to the locoregional lymph drainage pathways varies between tomotherapy plans and conventional 3D- tangentially arranged fields. Future studies will show whether this has an influence on loco-regional spread. In particular, it must be clarified whether different irradiation techniques should have implications for the target volume definition guidelines.

## Data Availability

The data supporting the conclusions of this article are included within the article.

## Abbreviations

BCS: Breast-conserving surgery
CRT: Conventional radiotherapy
DIBH: Deep inspiration breath hold
Dmean: Mean Dose
Gy: Gray
IGRT: Image-guided radiotherapy
IMLNs: Internal mammary lymph nodes
IMRT: Intensity-modulated radio therapy
RT: Radiotherapy
TF: Tangential field
TT: Tomotherapy

## References

[1] Gradishar WJ, Anderson BO, Balassanian R, Blair SL, Burstein HJ, Cyr A (2016). Invasive breast Cancer version 1.2016, NCCN clinical practice guidelines in oncology. J Natl Compr Cancer Netw.

[2] Sautter-Bihl ML, Sedlmayer F, Budach W, Dunst J, Feyer P, Fietkau R (2014). DEGRO practical guidelines: radiotherapy of breast cancer III--radiotherapy of the lymphatic pathways. Strahlenther Onkol.

[3] Overgaard M, Hansen PS, Overgaard J, Rose C, Andersson M, Bach F (1997). Postoperative radiotherapy in high-risk premenopausal women with breast cancer who receive adjuvant chemotherapy. Danish breast Cancer cooperative group 82b trial. N Engl J Med.

[4] Overgaard M, Jensen MB, Overgaard J, Hansen PS, Rose C, Andersson M (1999). Postoperative radiotherapy in high-risk postmenopausal breast-cancer patients given adjuvant tamoxifen: Danish breast Cancer cooperative group DBCG 82c randomised trial. Lancet.

[5] Darby S, McGale P, Correa C, Taylor C, Arriagada R, Early Breast Cancer Trialists’ Collaborative G (2011). Effect of radiotherapy after breast-conserving surgery on 10-year recurrence and 15-year breast cancer death: meta-analysis of individual patient data for 10,801 women in 17 randomised trials. Lancet.

[6] Giuliano AE, McCall L, Beitsch P, Whitworth PW, Blumencranz P, Leitch AM (2010). Locoregional recurrence after sentinel lymph node dissection with or without axillary dissection in patients with sentinel lymph node metastases: the American College of Surgeons oncology group Z0011 randomized trial. Ann Surg.

[7] Whelan TJ, Olivotto IA, Parulekar WR, Ackerman I, Chua BH, Nabid A (2015). Regional nodal irradiation in early-stage breast Cancer. N Engl J Med.

[8] Poortmans PM, Collette S, Kirkove C, Van Limbergen E, Budach V, Struikmans H (2015). Internal mammary and medial supraclavicular irradiation in breast Cancer. N Engl J Med.

[9] Thorsen LB, Thomsen MS, Berg M, Jensen I, Josipovic M, Overgaard M (2014). CT-planned internal mammary node radiotherapy in the DBCG-IMN study: benefit versus potentially harmful effects. Acta Oncol.

[10] Leite ET, Ugino RT, Santana MA, Ferreira DV, Lopes MR, Pelosi EL (2016). Incidental irradiation of internal mammary lymph nodes in breast cancer: conventional two-dimensional radiotherapy versus conformal three-dimensional radiotherapy. Radiol Bras.

[11] Belkacemi Y, Allab-Pan Q, Bigorie V, Khodari W, Beaussart P, Totobenazara JL (2013). The standard tangential fields used for breast irradiation do not allow optimal coverage and dose distribution in axillary levels I-II and the sentinel node area. Ann Oncol.

[12] Jung J, Kong M, Kim SS, Yoon WS (2016). Coverage of axillary lymph nodes with tangential breast irradiation in Korea: a multi-institutional comparison study. Int J Breast Cancer.

[13] Alco G, Igdem SI, Ercan T, Dincer M, Senturk R, Atilla S (2010). Coverage of axillary lymph nodes with high tangential fields in breast radiotherapy. Br J Radiol.

[14] Reed DR, Lindsley SK, Mann GN, Austin-Seymour M, Korssjoen T, Anderson BO (2005). Axillary lymph node dose with tangential breast irradiation. Int J Radiat Oncol Biol Phys.

[15] Jagsi R, Chadha M, Moni J, Ballman K, Laurie F, Buchholz TA (2014). Radiation field design in the ACOSOG Z0011 (Alliance) trial. J Clin Oncol.

[16] Joo JH, Kim SS, Ahn SD, Kwak J, Jeong C, Ahn SH (2015). Cardiac dose reduction during tangential breast irradiation using deep inspiration breath hold: a dose comparison study based on deformable image registration. Radiat Oncol.

[17] Borca VC, Franco P, Catuzzo P, Migliaccio F, Zenone F, Aimonetto S (2012). Does TomoDirect 3DCRT represent a suitable option for post-operative whole breast irradiation? A hypothesis-generating pilot study. Radiat Oncol.

[18] Franco P, Catuzzo P, Cante D, La Porta MR, Sciacero P, Girelli G (2011). TomoDirect: an efficient means to deliver radiation at static angles with tomotherapy. Tumori.

[19] Duma MN, Molls M, Trott KR (2014). From heart to heart for breast cancer patients - cardiovascular toxicities in breast cancer radiotherapy. Strahlenther Onkol.

[20] Lee JW, Hong S, Choi KS, Kim YL, Park BM, Chung JB (2008). Performance evaluation of field-in-field technique for tangential breast irradiation. Jpn J Clin Oncol.

[21] RTOG (2014). RTOG: Breast cancer atlas for radiation therapy planning: consensus definitions.

[22] Krasin M, McCall A, King S, Olson M, Emami B (2000). Evaluation of a standard breast tangent technique: a dose-volume analysis of tangential irradiation using three-dimensional tools. Int J Radiat Oncol Biol Phys.

[23] Francolini G, Thomsen MS, Yates ES, Kirkove C, Jensen I, Blix ES (2017). Quality assessment of delineation and dose planning of early breast cancer patients included in the randomized Skagen trial 1. Radiother Oncol.

[24] Aristei C, Chionne F, Marsella AR, Alessandro M, Rulli A, Lemmi A (2001). Evaluation of level I and II axillary nodes included in the standard breast tangential fields and calculation of the administered dose: results of a prospective study. Int J Radiat Oncol Biol Phys.

[25] Borm KJ, Oechsner M, Combs SE, Duma MN (2018). Deep-inspiration breath-hold radiation therapy in breast Cancer: a word of caution on the dose to the axillary lymph node levels. Int J Radiat Oncol Biol Phys.

[26] De Santis MC, Bonfantini F, Dispinzieri M, Meroni S, Diletto B, Mantero ED (2016). Axillary coverage by whole breast irradiation in 1 to 2 positive sentinel lymph nodes in breast cancer patients. Tumori.

[27] Kataria T, Bisht SS, Gupta D, Goyal S, Jassal K, Abhishek A (2013). Incidental radiation to axilla in early breast cancer treated with intensity modulated tangents and comparison with conventional and 3D conformal tangents. Breast.

[28] Lee J, Kim SW, Son SH (2016). Dosimetric evaluation of incidental irradiation to the axilla during whole breast radiotherapy for patients with left-sided early breast cancer in the IMRT era. Medicine (Baltimore).

[29] Zhang L, Yang ZZ, Chen XX, Tuan J, Ma JL, Mei X (2015). Dose coverage of axillary level I-III areas during whole breast irradiation with simplified intensity modulated radiation therapy in early stage breast cancer patients. Oncotarget.

[30] Larson D, Weinstein M, Goldberg I, Silver B, Recht A, Cady B (1986). Edema of the arm as a function of the extent of axillary surgery in patients with stage I-II carcinoma of the breast treated with primary radiotherapy. Int J Radiat Oncol Biol Phys.

[31] Withers HR, Peters LJ, Taylor JM (1995). Dose-response relationship for radiation therapy of subclinical disease. Int J Radiat Oncol Biol Phys.

[32] Marks LB (1990). A standard dose of radiation for “microscopic disease” is not appropriate. Cancer.

[33] Withers HR, Suwinski R (1998). Radiation dose response for subclinical metastases. Semin Radiat Oncol.

[34] Setton J, Cody H, Tan L, Morrow M, Hudis C, Catalano J (2012). Radiation field design and regional control in sentinel lymph node-positive breast cancer patients with omission of axillary dissection. Cancer.

[35] Gentilini O, Botteri E, Rotmensz N, Da Lima L, Caliskan M, Garcia-Etienne CA (2009). Conservative surgery in patients with multifocal/multicentric breast cancer. Breast Cancer Res Treat.

[36] Sixel KE, Aznar MC, Ung YC (2001). Deep inspiration breath hold to reduce irradiated heart volume in breast cancer patients. Int J Radiat Oncol Biol Phys.

[37] Haffty BG, Whelan T, Poortmans PM (2016). Radiation of the internal mammary nodes: is there a benefit?. J Clin Oncol.

